# Preserving plant samples from remote locations for detection of RNA and DNA viruses

**DOI:** 10.3389/fmicb.2022.930329

**Published:** 2022-08-25

**Authors:** Islam Hamim, Jon Y. Suzuki, Wayne B. Borth, Michael J. Melzer, Marisa M. Wall, John S. Hu

**Affiliations:** ^1^Department of Plant Pathology, Bangladesh Agricultural University, Mymensingh, Bangladesh; ^2^USDA-ARS, Daniel K. Inouye U.S. Pacific Basin Agricultural Research Center, Hilo, HI, United States; ^3^Department of Plant and Environmental Protection Sciences, University of Hawaii at Manoa, Honolulu, HI, United States

**Keywords:** sample preservation, remote sampling, plant virus, detection, characterization

## Abstract

Viral diseases in plants have a significant impact on agricultural productivity. Effective detection is needed to facilitate accurate diagnosis and characterization of virus infections essential for crop protection and disease management. For sensitive polymerase chain reaction (PCR)-based methods, it is important to preserve the integrity of nucleic acids in plant tissue samples. This is especially critical when samples are collected from isolated areas, regions distant from a laboratory, or in developing countries that lack appropriate facilities or equipment for diagnostic analyses. RNA*later*^®^ provides effective, reliable sample storage by stabilizing both RNA and DNA in plant tissue samples. Our work indicated that total RNA or DNA extracted from virus-infected leaf samples preserved in RNA*later*^®^ was suitable for reverse transcription polymerase chain reaction (RT-PCR), PCR, Sanger sequencing, high-throughput sequencing (HTS), and enzyme-linked immunosorbent assay (ELISA)-based diagnostic analyses. We demonstrated the effectiveness of this technology using leaf tissue samples from plants with virus symptoms grown in farmers’ fields in Bangladesh. The results revealed that RNA*later*^®^ technology was effective for detection and characterization of viruses from samples collected from remote areas and stored for extended periods. Adoption of this technology by developing countries with limited laboratory facilities could greatly increase their capacity to detect and diagnose viral infections in crop plants using modern analytical techniques.

## Introduction

Studies have shown that leaf tissue samples of virus-infected plants can degrade rapidly during transit or storage and require immediate appropriate sampling and preservation (Wang et al., 2018a). This is needed to obtain DNA and RNA of sufficient quality and quantity, the most important step for subsequent reliable molecular detection and characterization of plant viruses (Huang et al., 2017; Mäki et al., 2017; Patel et al., 2017). Sample preservation for virus detection usually involves freezing plant tissues with dry ice or liquid nitrogen and then either storing the frozen samples in an ultracold freezer or shipping them on dry ice (Prendini et al., 2002). The cost of shipping with dry ice from remote areas is high and can make sample preservation an obstacle in developing countries. RNA and DNA can be quickly degraded by endogenous nucleases and by oxidative processes. Degraded DNA is of limited use because large DNA fragments may be required for downstream molecular analyses (Sirakov, 2016). RNA can also be degraded by cellular and environmental endogenous ribonucleases if transit or storage conditions are not ideal, or are of long duration (Fleige and Pfaffl, 2006; Relova et al., 2018).

RNAl*ater*^®^ is a highly effective fixative for preserving biological materials (Bennike et al., 2016; Kruse et al., 2017). It is a commercially available reagent that acts as a nucleic-acid-preserving buffer, containing high concentrations of quaternary ammonium sulfates and cesium sulfates that inhibit degradation of RNA or DNA by RNases and DNases. RNAl*ater*^®^ can preserve RNA and DNA in plant tissue at room temperature for several weeks and is recommended for PCR and genome sequencing analyses (Yockteng et al., 2013). To date, detection and characterization of plant viruses from leaf tissue stored in RNAl*ater*^®^ has not been detailed. We assessed the efficacy of collecting papaya (*Carica papaya*), banana (*Musa paradisiaca*), and taro (*Colocasia esculenta*) leaves with virus-like symptoms in RNAl*ater*^®^ to protect their RNA and DNA. We then analyzed the tissues using enzyme-linked immunosorbent assay (ELISA), polymerase chain reaction (PCR), complementary DNA (cDNA) synthesis, reverse transcription (RT)-PCR, single-tube nested PCR (STNP), and high-throughput sequencing (HTS). We identified complete and partial genomes of viruses and subviral agents using this approach. These findings could be useful in surveillance programs during virus outbreaks in agricultural systems of developing countries.

## Materials and methods

### Effect of extended storage times in RNA*later^®^* on nucleic acid quality, quantity and virus detection and characterization by PCR and ELISA

Papaya leaf samples with typical virus-like symptoms such as mosaic, ringspots, and leaf distortions as well as asymptomatic leaf samples, were collected from the island of Oahu, Hawaii. The samples were cut into small pieces and 500 mg added to 2 ml of RNAl*ater*^®^ solution (Qiagen, Germany). After 7, 15, or 30 days at room temperature, immersed leaf tissues were kept at 4°C for 3 days before being transferred to 20°C for 1 week and then stored at-80°C for 1 month.

Leaf samples selected for plant virus detection were cleaned and dried with a paper towel, and then ground into powder with liquid nitrogen in a sterile mortar and pestle. Total RNAs were extracted from 100 mg of the sample with the RNeasy^®^ Plant Mini Kit (Qiagen Inc., Germany). The RNAs were eluted in 80 μl of RNase-free water and stored at-80°C for later use. Yield and quality of the total RNAs were assayed using 1% agarose gel electrophoresis and spectrophotometry.

Papaya total RNA samples were tested for viruses by RT-PCR assays using universal potyvirus-specific nuclear inclusion body (NIb) primers (Zheng et al., 2010), papaya ringspot virus (PRSV)-specific coat protein (CP) primers (Bateson et al. 1994) and PRSV-specific STNP assay (Hamim et al., 2018), resulting in amplicons of 350 bp, 905 bp and 128 bp, respectively. The reaction mixture consisted of 2 μl RNA, 1 μl random hexamer primers (50 μg/ml) and 6.5 μl RNase-free H_2_O. The reaction was incubated for 10 min at 72°C and quickly chilled on ice. A cocktail of 5 μl dNTP mixture (2.5 mmol/l each), 4 μl 5 × MMLV reaction buffer, 1 μl MMLV reverse transcriptase (200 U/μl) and 0.5 μl RNase inhibitor (40 U/μl) was then added (Promega, United States). The RT reaction was incubated at 25°C for 10 min, and then 42°C for 50 min. Reactions were chilled on ice and stored at-20°C prior to potyvirus NIb (Zheng et al., 2010) or PRSV-CP (Bateson et al. 1994) RT-PCR assay and PRSV-specific STNP (Hamim et al., 2018). Specific primer pairs reported by Noa-Carrazana et al. (2007; Table 1) were used to amplify overlapping DNA fragments corresponding to a full-length PRSV genome of a Hawaii isolate from papaya leaf samples.

**Table 1:** Primer pairs used in this study for detection and genome characterization of plant viruses from leaf samples stored in RNA*later*^®^.

Primer name	Sequence	Virus isolate	Target genome	Amplified product (nt)	Reference
12-FP	AAATAAAACATCTCAACACAACATAATCAAAAG	PRSV HA	1–1,780	1,780	Noa-Carrazana et al., 2007
50-RP	GCCGTAGAAAAGCCCTATTAAAACC				
27-FP	GAAGAAGCATGGACAATACAGATTGGGG	PRSV HA	708–2,303	1,595	Noa-Carrazana et al., 2007
28-RP	GGGTTGAAGTGAGCTTTCCCAGAACGCT				
49-FP	AGAGTAATTGACGAAGTTGGTGAGGA	PRSV HA	1917–3,567	1,650	Noa-Carrazana et al., 2007
51-RP	GCGTAGGTTTTTTCCACAGC				
52-FP	GCAGTTGACGGATGAAGGTCTACTCA	PRSV HA	3,482–4,631	1,149	Noa-Carrazana et al., 2007
53-RP	GAGGTCTGGTGGGCTCTATT				
54-FP	GTCTTTAGATGACATTCGGGATTTCTAT	PRSV HA	4,279–6,305	2,026	Noa-Carrazana et al., 2007
118-RP	GTGAAACCGCATACAATCGCATCCT				
26-FP	CATTTTAAAGCCATGACAAGTTGCACTGG	PRSV HA	5,972–7,542	1,570	Noa-Carrazana et al., 2007
55-RP	GCTCCAGTGTTTGCTCCAAGTTAAAT				
56-FP	GATTGATGATTTAACTTGGA	PRSV HA	7,508–9,389	1881	Noa-Carrazana et al., 2007
59-RP	GACACATCATTTCCGTCACT				
3-FP	GACCATGGTCCTAGAATGAAGCTGTGGATG	PRSV HA	9,249–10,320	1,071	Noa-Carrazana et al., 2007
11-RP	TTTTTTTTCTCTCATTCTAAGAGGCTC				
S11F	CTCAACACAACATAATTCAAAGCAATCTACA	PRSV BD-1	1–1,254	1,254	Hamim et al. 2019a
S11R	TCGAAGTTGCCTGCTTGATACCA				
S12F	GAAATTTCTGTCCCACTCTTGGTGATTA	PRSV BD-1	714–1,869	1,155	Hamim et al. 2019a
S12R	TACACGTTATCTTGTGACAAGG				
S13F	CACGGGTCGAGTGGTCTAATTTACA	PRSV BD-1	1,551–2,946	1,395	Hamim et al. 2019a
S13R	TCTTTTGTTTGTGGTCAACGAGAATCCTT				
S14F	AAC AGA TGG AGC ATA TTG A	PRSV BD-1	2,494–4,363	1,869	Hamim et al. 2019a
S14 R	TCA ATT GGT CCG CGA TTA AT				
S15F	AGA GGC TGT TGA AAA AAC CTA C	PRSV BD-1	3,521–5,987	2,466	Hamim et al. 2019a
S15R	GCGA ATT CAA ACC AGT ACA ACT GGT CAT TG				
S16F	CATTAAGGATGTTCC AGA GAG GTT GTA	PRSV BD-1	5,750–7,636	1866	Hamim et al. 2019a
S16R	CGA CTC CCA CTC TGC TCT ATA ACA T				
S17F	GATTTCGGGAGTCACTGGATCTCA ACT A	PRSV BD-1	7,317–9,561	2,244	Hamim et al. 2019a
S17R	ACT GTG ATT GAG TGG CAC GAG TGT				
S18F	ACG AGT CAA AAA GCT TAG GAG ATT ACC AA	PRSV BD-1	9,172–10,300	1,128	Hamim et al. 2019a
S18R	CTC TCA TTC TAA GAG GCT CAG ATA G				
S21F	CAT AAA CGA TGC CGA CTC AAC AC	PRSV BD-2	1–904	904	Hamim et al. 2019a
S21R	CCA ACT TCC AGT GCC AGA T				
S22F	ACACCATCTGAGATTGATTCAATCG	PRSV BD-2	517–2,051	1,534	Hamim et al. 2019a
S22R	AAAACTCTGTTCAGTTGATCAAGCA				
S23F	AAGCCGAAGTTTGGTGATAAAATAG	PRSV BD-2	1,606–3,194	1,588	Hamim et al. 2019a
S23R	TTTCTGAGCAAATCTCGAAGTTCGT				
S24F	AAGACTCCAACCAAGAATCACAT	PRSV BD-2	2,656–4,334	1,678	Hamim et al. 2019a
S24 R	TTGTCCTCATAGAAGTCCTGAATGTC				
S25F	CAGAGAGATTTTGATCGGTTAGAA	PRSV BD-2	3,985–5,965	1,980	Hamim et al. 2019a
S25R	TTTCACTCTCAATCAGTTTGTCG				
S26F	TGCACTGTACGGCAAGCAC	PRSV BD-2	5,524–7,400	1,876	Hamim et al. 2019a
S26R	AGTGGACTACCACAATCACCATCAT				
S27F	GGATAAAGTTTGCATGATAGGTG	PRSV BD-2	7,257–9,422	2,165	Hamim et al. 2019a
S27R	TTAACATCTCTATCTCTCTCTCCA				
S28F	GGCTCCTTATGTCTCTGAAGTT	PRSV BD-2	9,069–10,325	1,256	Hamim et al. 2019a
S28R	CTCTCATTCTAAGAGGCTCAAATATCA				
MKBEGAF4	ATATCTGCAGGGNAARATHTGGATGGA	Begomovirus	646–1,945	1,300	Hamim et al., 2019b
MKBEGAR5	TGGACTGCAGACNGGNAARACNATGTGGGC				
PA	TAATATTACCKGWKGVCCSC	ToLCBV	2,754–1,417	1,424	Hamim et al., 2019b
ToLCBV39R2	CAAGCCGAGAATGGCGTGTATATG				
ToLCBV DNA2F	AGCTGCAGTGATGAGTTCC	ToLCBV	1,440–528	1,849	Hamim et al., 2019b
PB	TGGACYTTRCAWGGBCCTTCACA				
ToLCBVDNA A1F	ACCGGATGGCCGCGATTT	ToLCBV	1–1,417	1,417	Hamim et al., 2019b
ToLCBV39R2	CAAGCCGAGAATGGCGTGTATATG				
ToLCBV DNA2F	AGCTGCAGTGATGAGTTCC	ToLCBV	1,460–2,760	1,321	Hamim et al., 2019b
ToLCBV39R1	AATATTAGTGCGGATGGCCGCTTT				
Beta 01	GGTACCACTACGCTACGCAGCAGCC	Betasatellite	1,303–1,327	1,367	Hamim et al., 2019b
Beta 02	GGTACCTACCCTCCCAGGGGTACAC		1,309–1,284		
ToLCBbetaPaF	ACCGTGGGCGAGCGGGGTTTTTGGC	ToLCB	1–1,367	1,367	Hamim et al., 2019b
ToLCBbetaPaR	AATATTAGAACGTGGGCGAGCTAAG				
J1F	CTATGTACTGGGCAAGATTTGGATG	ToLCJoV	647–1,779	1,132	Hamim et al., 2020
J1R	CAAAGGGACTGGCAATCAAATACG				
J2F	CTAATATTACCGGATGGCCGCGAT	ToLCJoV	2,763–1,441	1,322	Hamim et al., 2020
J2R	CAGGCCGAGAATGGCGTGTTTATC				
J3F	AGCTGCAGTGATGGGTTCC	ToLCJoV	1,442–529	913	Hamim et al., 2020
J3R	TGGACCTTACATGGGCCTTCACA				
J4F	ACCGGATGGCCGCGATTT	ToLCJoV	1–1,441	1,441	Hamim et al., 2020
J4R	CAGGCCGAGAATGGCGTGTTTATC				
J5F	AGCTGCAGTGATGGGTTCC	ToLCJoV	1,422–2,762	1,340	Hamim et al., 2020
J5R	AATATTAGTGCGGATGGCCGCTTT				
B1F	GGTACCACTACGCTACGCAGCAGCC	ToLCJoB	1,301–1,325	1,365	Hamim et al., 2020
B1R	GGTACCTACCCTCCCAGGGGTACAC		1,306–1,282		
B2F	ACCGTGGGCGAGCGGGGTCTTTGGC	ToLCJoB	1–1,365	1,365	Hamim et al., 2020
B2R	AATATTAGAACGTGGGCGAGCTAGG				
B3F	GATTTGTTTATCTGACTGAAACTCC	ToLCJoB	1,197–799	967	Hamim et al., 2020
B3R	GTG ACCTTCTCTCTCCTAAAAACT				
ND1F	CTATGTGCTGGGGAAGATATGGATGGA	ToLCNDV DNA-A	621–1,922	1,301	Hamim et al., 2020
ND1R	GGATAGTAGGACAGGCAAAACAATGTG				
ND2F	TAATATTACCGAATGGCCGC	ToLCNDV DNA-A	2,741–1,415	1,326	Hamim et al., 2020
ND2R	CGAGCAGAGAGTGGCGTATATACC				
ND3F	ATCCGCAGTGATGTATTCC	ToLCNDV DNA-A	1,416–501		Hamim et al., 2020
ND3R	CACCTTGCAAGGGCCTTCACA				
ND4F	ACCGAATGGCCGCGCAAAT	ToLCNDV DNA-A	1–1,415	1,415	Hamim et al., 2020
ND4R	CGAGCAGAGAGTGGCGTATATACC				
ND5F	ATCCGCAGTGATGTATTCC	ToLCNDV DNA-A	1,416–2,740	1,325	Hamim et al., 2020
ND5R	AATATTATACGAATGGCCGCTTT				
ND6F	ACCCGTAACGATCTTGAACTTTGTCC	ToLCNDV DNA-A	1,544–1,569		Hamim et al., 2020
ND6R	GGTACCATATTTGGCAATAGGTCCGAA		1,520–1,546		
ND7F	CAATATACGCGTAAGGAAATATGTG	ToLCNDV DNA-A	623–1,517	894	Hamim et al., 2020
ND7R	AATCATGGGCTAGCAGATCG				
ND8F	ACCGAAAGGCCGCGAAAAT	ToLCNDV DNA-B	1–1,458	1,458	Hamim et al., 2020
ND8R	AAC CTG AGC GGA CTG GAC GAT T				
ND9F	AATATTATACGAAAGGCCGCTT	ToLCNDV DNA-B	1,437–2,688	1,251	Hamim et al., 2020
ND9R	AATCGTCCAGTCCGCTCAGGTT				
ND10F	TGCAATCTCTTCCCTTGTGATA	ToLCNDV DNA-B	1,375–767		Hamim et al., 2020
ND10 R	TTAGCTTGATGTACGAACGAA				

PRSV-specific ELISA was performed according to the manufacturer’s (Agdia, United States) instructions, and absorbance at 405 nm measured with a Bio-Rad Model 680 microplate reader (BioRad, United States). Absorbance ratios of values obtained from samples derived from symptomatic and asymptomatic tissues were calculated from mean values of absorbance for each extract dilution to set a positive/negative threshold (Sreenivasulu and Gopal 2010).

### Remote collection of papaya, banana, and taro leaf samples in Bangladesh

Leaf samples of papaya, banana, and taro with and without virus-like symptoms were collected in Bangladesh during a field survey from December 2016 to January 2017 and immediately stored in RNA*later*^®^ solution. The samples were then shipped at room temperature to the University of Hawaii at Mānoa’s plant virology laboratory under USDA PPQ 526 permits P526P-16-03156 and P526P-16-03662. The delivery service and quarantine procedures took 1 week to complete. Upon arrival, all samples were stored at 4°C for 3 days, followed by −20°C for 1 week, and − 80°C until total RNA extractions were conducted.

### Efficacy of RNA*later^®^* for determining whole-genome sequences of PRSV isolates by HTS from papaya leaf samples collected in Bangladesh

PRSV-specific STNP (Hamim et al., 2018), and ELISA (Agdia Inc., United States) analyses were carried out on 11 papaya leaf samples collected from Bangladesh and preserved in RNA*late*r^®^ to confirm the presence of PRSV. RNA*later*^®^ was removed from the plant tissue as described above. Total RNAs were extracted from leaf samples using the RNeasy^®^ Plant Mini Kit, and cDNAs synthesized according to the method described previously. For HTS analyses, PRSV-positive leaf samples were divided into two separate composite samples, pap-1 and pap-2, at Foundation Plant Services, Davis, California. Composite samples, prepared by combining 10-μl aliquots of total RNA from each subsample, were subjected to ribosomal RNA (rRNA) depletion and cDNA library synthesis using the Ribo-Zero^®^ Plant Kit (Illumina, Inc., United States) and TruSeq^®^ Stranded Total RNA (Illumina, Inc., United States), respectively (Hamim, 2019; Hamim et al., 2019a). Sequencing was performed using the Illumina^®^ NextSeq 500 platform, and the raw HTS reads analyzed at Foundation Plant Services (Al Rwahnih et al., 2018). Employing the CLC Bio Genomic Workstation (v8.5.1; Qiagen, Hilden, Germany), Illumina reads were adapter-trimmed and then *de novo* assembled into contigs of at least 200 bp in length. The list of contigs was created by comparing contig sequences to the viral genome section of NCBI RefSeq^1^ using tBLASTx software (v. 2.4.0). Candidates included contigs that matched viral genomes and had a total E-value of 10^−4^ or below. To provide the annotation finally required for viral agent identification, the narrowed list of viral hits was then evaluated against the complete nonredundant GenBank databases using BLASTx to match sequences against protein (nr) and BLASTn to compare sequences against nucleotides (nt).

Full-length sequences of PRSV genomes BD-1 and BD-2 obtained by HTS from composite samples pap-1 and pap-2, respectively, were verified by direct sequencing of PCR-amplified overlapping DNA fragments using virus-specific primers designed from the HTS-derived sequences (Table 1). PCR amplifications were performed on cDNA synthesized from total RNA as described above and amplified using the following conditions: 4 min at 94°C; 35 cycles of 1 min at 94°C, 1 min at 50 to 65°C depending on the primers, and 2 min at 72°C; with a final elongation at 72°C for 7 min (Hamim, 2019; Hamim et al., 2019a). The PCR products were resolved on 1% agarose gel in 1X TAE buffer. Gels were stained with ethidium bromide and visualized in a UVP transilluminator. PCR products of the predicted size were purified, cloned into the PGEM-T Easy^®^ cloning vector (Promega, United States), and sequenced at the University of Hawaii’s Advanced Genomic and Sequencing Services and at Genewiz, in California.

### Efficacy of RNA*later^®^* for detection of isolates of PRSV and Begomoviruses from papaya leaf samples collected from different remote regions in Bangladesh

The presence of specific PRSV isolates was detected by RT-PCR assays from RNA*later*^®^-preserved papaya leaf samples with virus-like symptoms: mosaic, ring spots, leaf curl and leaf distortion collected from eight geographical districts in Bangladesh (Table 3). Total RNAs were extracted from the samples using the RNeasy^®^ Plant Mini Kit, and cDNAs synthesized as reported (Hamim, 2019; Hamim et al., 2019a).

Total DNAs were extracted from 100 mg of the papaya leaf samples from Bangladesh stored in RNA*later*^®^ with the DNeasy^®^ Mini extraction kit (Qiagen Inc., Germany). The DNAs were eluted in 50 μl of DNase-free water and stored at-20°C for later use. Yield and quality of the total RNAs were assayed using spectrophotometry (Thermo Fisher Scientific, Waltham, MA, United States). DNA extracted from papaya leaf samples was used to test for the presence of begomoviruses using PCR with begomovirus-specific primer pairs (Table 1), accompanied by Sanger sequencing (Hamim et al., 2020). From these analyses, full-length genome sequences of specific begomoviruses as well as the cognate betasatellites or associated components and defective betasatellites were obtained by direct sequencing of PCR-amplified overlapping DNA fragments using virus-specific primers (Table 1). In addition, RNA*later*^®^-stored papaya leaf samples were used to examine the geographic distribution of various begomoviruses by PCR using virus-specific primers.

### Efficacy of RNA*later^®^* for detection of viruses in taro and banana leaf samples collected from Bangladesh

Total RNA was extracted from taro leaf samples in RNA*later^®^* using the RNeasy Plant Mini Kit and synthesis of cDNA was performed using M-MLV reverse transcription (Promega, United States; Wang et al., 2017). A universal potyvirus-specific ELISA (Agdia Inc., USA) and a universal potyvirus-specific RT-PCR (Zheng et al., 2010) were used to test two samples with feathery mottling and distorted leaves, and one asymptomatic sample. RT-PCR with dasheen mosaic virus (DsMV) CI-specific primers and a DsMV-specific triple-antibody sandwich ELISA (Agdia Inc., United States; Wang et al., 2017) were used to confirm DsMV in taro leaf samples.

We used the CTAB method to isolate total DNA from 100 mg banana leaf samples with banana bunchy top virus (BBTV) symptoms kept in RNA*later*^®^ using CTAB method and were tested by PCR using BBTV-Rep gene-specific primers and CP-specific primers BBTV-HAF1 and BBTV-HAR1 (Hamim et al., 2017). Six samples were also tested for BBTV using a BBTV-specific antibody in a triple-antibody sandwich-ELISA (Agdia, Elkhart, Inc.).

## Results

### Effect of extended storage times in RNA*later^®^* on detection and characterization of RNA viruses

**Figure 1:**
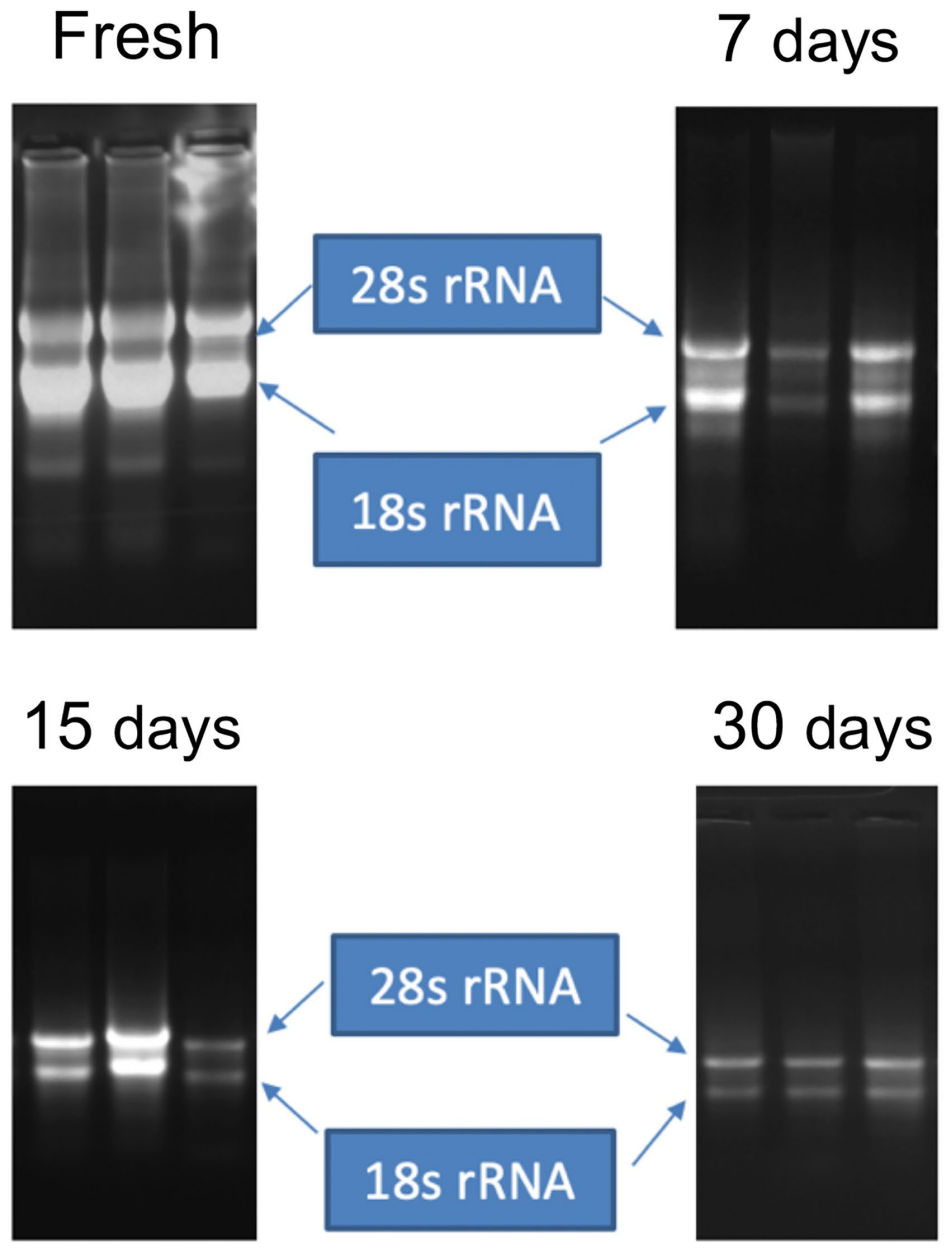
Gel electrophoresis of total RNA extracted from fresh leaves or leaves harvested and stored in RNA*later*^®^ for 7, 15 or 30 days. Bands corresponding to intact ribosomal RNA (rRNA) species are shown.

**Figure 2:**
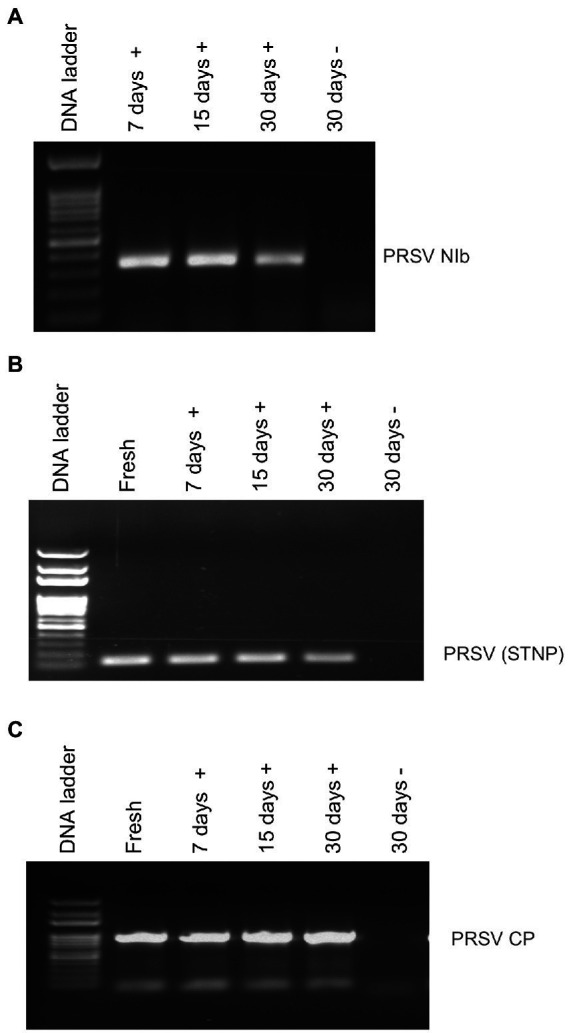
Analysis of RT-PCR products from reactions using **(A)** potyvirus-specific nuclear inclusion body (NIb) primers, **(B)** PRSV coat protein (CP) primers or **(C)** PRSV-specific single-tube nested PCR (STNP) and total RNA from symptomatic (+) or asymptomatic (−) fresh papaya leaves or leaves stored in RNA*later*^®^ for various periods.

**Figure 3:**
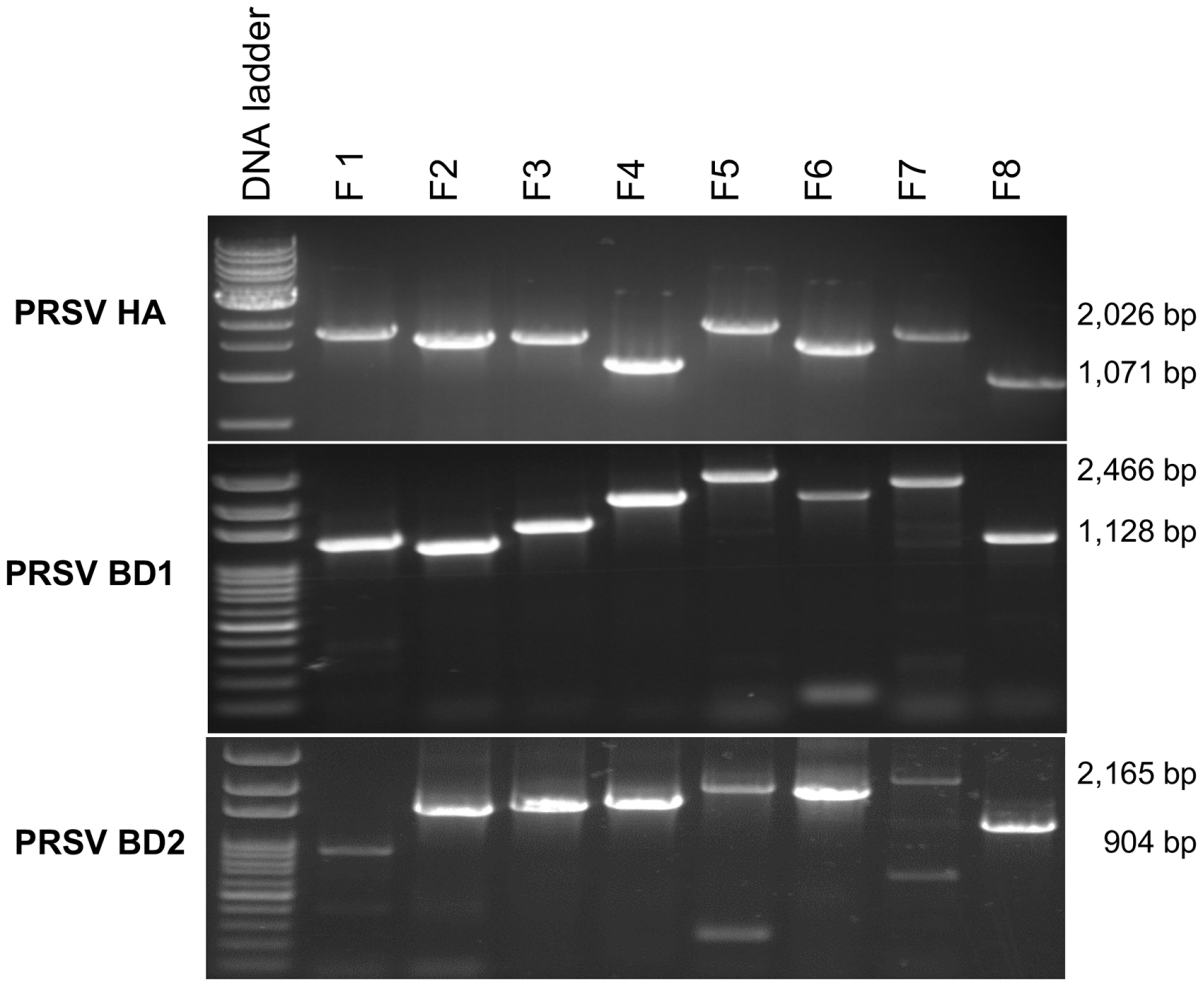
Analysis of overlapping RT-PCR DNA fragments (F1 to F8) covering full-length PRSV genomes using published PRSV primer sequences for Hawaii isolate (HA) or using primers designed based on HTS analysis for PRSV Bangladesh isolates BD1 and BD2 and total RNA from symptomatic papaya leaf samples stored in RNA*later*^®^ for one-month. Size ranges of PCR fragments are shown at right.

Average RNA yields obtained from virus-infected papaya leaves collected on Oahu, Hawaii, and stored for 7, 15, and 30 days in RNA*later*^®^ were 315.17 ± 62.23 ng/μl, 355.1 ± 86.5 ng/μl and 211.03 ± 61.03 ng/μl, respectively, compared to 670.17 ± 216.18 ng/μl total RNA from fresh samples. A 260/280 ratio greater than 2.0 was obtained for RNAs isolated from both RNA*later*^®^-stored and fresh papaya leaf samples. Gel electrophoresis analysis of total RNA in agarose gels confirmed the presence of bands corresponding to the 28S and 18S ribosomal RNA (rRNA) for all storage times and for fresh samples, providing a qualitative assessment of the rRNA integrity in RNA*later*^®^-stored samples (Figure 1). The absence of smearing in the agarose gel indicated the total RNAs were intact and not degraded. Leaves stored in RNA*later*^®^ tested positive in potyvirus-specific RT-PCR with potyvirus NIb primers NIb2F and NIb3R (Figure 2A). Samples with virus-like symptoms stored in RNA*later*^®^ solution for different lengths of time, and fresh samples, were also positive in STNP reactions specific for PRSV, and RT-PCR reactions using primers specific for PRSV CP (Figures 2B,C, respectively) as were the ELISA assays. In addition, we successfully amplified eight overlapping DNA fragments (F1 to F8) correspond to 3′ to 5′ of the full-length PRSV genome of a Hawaiian PRSV isolate (HA) sequentially from a virus-infected papaya leaf sample that had been collected from Oahu, Hawaii and stored for 1 month in RNAlater^®^. These DNA fragments were amplified using previously reported primers (Figure 3; Table 1; Noa-Carrazana et al., 2007). Sizes of these amplified products varied from 1,071 to 2,026 bp.

Papaya leaf samples with virus-like symptoms collected from Bangladesh were used to study the efficacy of RNA*later*^®^ in combination with HTS technology for detecting viruses. The 11 samples stored in RNA*later*^®^, and tested with PRSV-specific STNP and ELISA were positive for PRSV. These samples were divided into two separate composite samples and full-length genomes of the two PRSV isolates, BD-1 and BD-2 identified by HTS (Table 2). The complete genome sequences of BD-1(MH444652) and BD-2 (MH397222) were verified by Sanger sequencing of overlapping DNA fragments obtained by RT-PCR using virus-specific primers and total RNA extracts from papaya leaf samples GAZ-52 and CD-129, respectively, which were stored in RNA*later*^®^ (Figure 3).

**Table 2:** Illumina^®^ NextSeq 500 RNA sequencing of whole papaya ringspot virus (PRSV) genomes from symptomatic papaya leaf samples collected in Bangladesh and stored in RNA*later*^®^.

PRSV Isolate	BD1	BD2
Total RNA composite	pap-1	pap-2
Total reads	80,648,570	61,307,471
Non-virus contigs	906	1,043
Plant virus contigs	134	79
PRSV-specific contigs	127	74
PRSV contig sequence lengths	200–10,282 nt	200–3,484 nt
Identical matches with reported isolates	52–100%	64–100%
Total genome length	10,300 nt	10,325 nt
GenBank accession no.	MH444652.1	MH397222.1

PRSV: Papaya ringspot virus.

The effectiveness of RNA*later*^®^ technology in routine RT-PCR assays for RNA viruses was also tested. Of the 118 papaya samples tested from eight different districts of Bangladesh, 22 were positive for PRSV BD-1, 81 were positive for PRSV BD-2, and both BD-1 and BD-2 were found in 13 of the samples (Table 3).

**Table 3:** Distribution and prevalence of PRSV isolates BD-1 and BD-2 based on RT-PCR analysis of papaya leaf samples obtained from different districts in Bangladesh and stored in RNA*late*r^®^.

Collection districts	Sample no.	PRSV BD-1 positive	PRSV BD-2 positive	PRSV BD-1 and BD-2 positive
Pabna	17	1	17	1
Rajshahi	5	0	3	0
Chapai Nawabganj	4	0	3	0
Chandpur	33	11	24	8
Munshiganj	9	2	5	1
Gazipur	22	8	15	3
Tangail	13	0	6	0
Mymensingh	15	0	8	0
Total	118	22	81	13

PRSV: Papaya ringspot virus.

**Figure 4:**
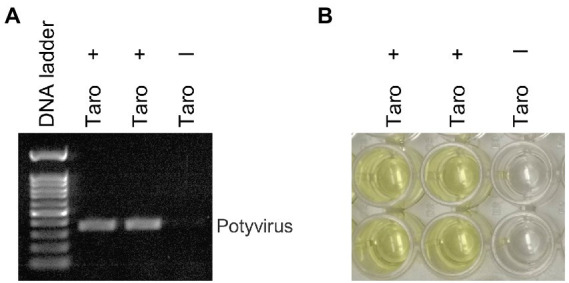
Analysis of taro leaf samples stored in RNA*later*^®^ for presence of potyvirus: **(A)** RT-PCR of total RNA from symptomatic (+) and asymptomatic (−) taro leaf samples, respectively using universal potyvirus primers; **(B)** potyvirus-specific ELISA of plant extracts from the same sample sources.

**Figure 5:**
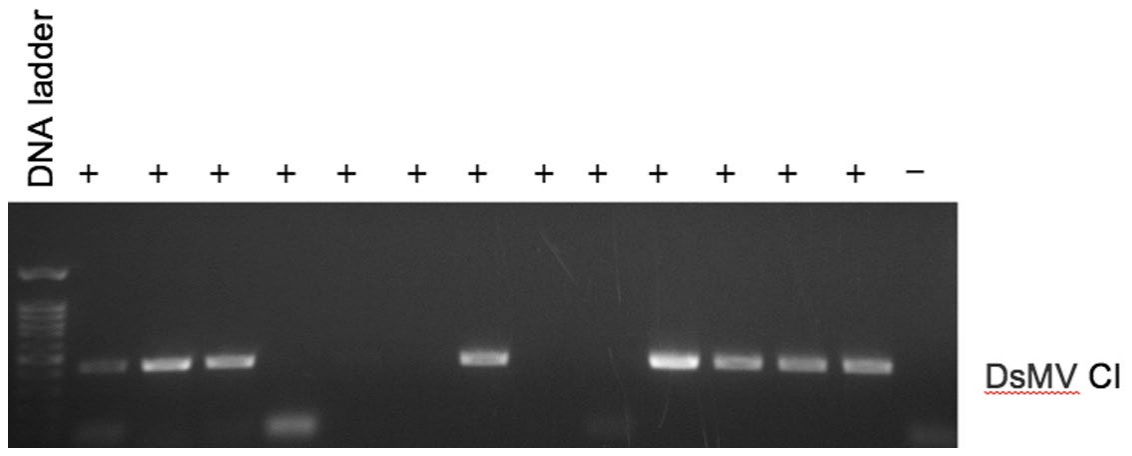
RT-PCR analysis of total RNA for detection of DsMV from symptomatic (+) taro leaf samples stored in RNA*later*^®^ or asymptomatic controls (−) using DsMV CI gene-specific primers.

We investigated the efficacy of RNA*later*^®^ technology for detecting dasheen mosaic virus (DsMV) in RNA*later*^®^-stored, symptomatic taro leaf samples. The two symptomatic samples tested positive for potyvirus with RT-PCR and ELISA, while an asymptomatic taro leaf sample tested negative (Figure 4). Sanger sequencing of the resulting PCR amplicons confirmed the presence of DsMV (accession MH036416; Wang et al., 2018b). DsMV CI-specific primers were used with RT-PCR to check for the presence of virus in total RNA extracts from 13 RNA*later^®^*-stored taro leaf samples (Figure 5; Wang et al., 2017). Eight of the samples produced target virus-specific bands. DsMV-specific triple-antibody sandwich-ELISA also confirmed that these eight samples were positive for DsMV.

### Effect of extended storage times in RNA*later^®^* on detection and characterization of DNA viruses

DNA extracted from RNA*later*^®^-stored papaya leaf samples was used to test for begomoviruses. A PCR test using a begomovirus-specific primer pair produced the predicted PCR product corresponding to the begomovirus DNA-A component in 43 of 51 samples examined. Of the 43 PCR products, Sanger sequencing identified 29 sequences corresponding to tomato leaf curl Bangladesh virus (ToLCBV; Table 4). The remainder were identified as being deriving from tomato leaf curl Joydebpur virus (ToLCJoV) and tomato leaf curl New Delhi virus (ToLCNDV; Table 4). We amplified and sequenced the full-length 2,760-bp genome of ToLCBV from RNA*later*^®^-stored Gaz17-Pap samples obtained from Gazipur, Bangladesh. A ToLCBV cognate betasatellite of 1,367 bp was also amplified from the DNA of the same Gaz17-Pap leaf sample (Table 5). Small defective betasatellites DefS39, DefS40, and Defs43 were also amplified from RNAlater^®^-stored, ToLCBV-positive samples Gaz19-Pap, Gaz-20-Pap, and Gaz21-Pap, respectively (Table 5). The complete 2,762-nucleotide sequence of ToLCJoV (MT127782) was amplified from RNA*later*^®^-stored papaya leaf sample J^−1^ (Table 5). Additionally, the betasatellite ToLCJoB (MT161673), 1,365 bp in length and associated with ToLCJoV, was obtained from the J1-Pap isolate (Table 5). The full-length DNA-A and DNA-B components of ToLCNDV were amplified and sequenced from the RNA*later*^®^-stored sample ND-71 (Table 5). These results indicated that DNA isolated from papaya tissues stored in RNA*later*^®^ solutions was able to support amplification of full-length plant DNA virus genomes.

**Table 4:** Distribution and prevalence of begomoviruses based on PCR analysis of papaya leaf samples obtained from various locations in Bangladesh and stored in RNA*later*^®^.

Collection districts	Sample no.	Begomovirus positive	ToLCBV Positive	ToLCJoV positive	ToLCNDV Positive
Pabna	11	7	3	3	1
ChapaiNwabgonj	4	4	1	0	3
Rajshahi	4	4	4	0	0
Gazipur	10	9	8	1	0
Tangail	4	4	3	1	0
Chandpur	10	10	5	5	0
Mymensingh	5	5	5	0	0
Total	51	43	29	10	4

ToLCBV: Tomato leaf curl Bangladesh virus.

ToLCJoV: Tomato leaf curl Joydebpur virus.

ToLCNDV: Tomato leaf curl New Delhi virus.

**Table 5:** GenBank **accessions of** Sanger sequence-derived nucleotide (nt) sequences representing begomovirus genome sequences from symptomatic papaya leaf samples collected in Bangladesh and stored in RNA*late*r^®^.

**Sample**	**ToLCBV**	**ToLCJoV**	**ToLCNDV**
Gazipur17-Pap	MH380003.1: 2,760 nt (98% nt identity to ToLCBV: KM383761)		
Gazipur17-Pap	Beta satellite MH397223.1:1,367 nt (92% nt identity to ToLCB: JN638434)		
Gazipur 19-Pap	Defective beta satellite (DefS)39 MH473589.1:564 nt (99% nt identical to corresponding regions of ToLCBV:MH380003)		
Gazipur 20-Pap	Defective beta satellite (DefS)40 MH507170.1:731 nt (94% nt identical to corresponding regions of ToLCBV:MH380003)		
Gazipur 21-Pap	Defective beta satellite (DefS)43 MH507169.1:738 nt (100% nt identical to corresponding regions of ToLCBV:MH380003)		
J1-Pap		MT127782.1:2,763 nt (95% nt identical to ToLCJV: KM383750)	
J1-Pap		Beta satellite MT161673.1: 1365 nt (95% nt identical to ToLCJoB: AJ966244.1)	
ND-71-Pap			DNA-A, MT161674.1: 2,688 nt (98% nt identical to ToLCNDV_DNA-A: KM383742) DNA-B, MT161675.1: 2,688 nt (93% nt identical to ToLCNDV_DNA-B: MG406983)

ToLCBV-Tomato leaf curl Bangladesh virus.

ToLCJoV-Tomato leaf curl Joydebpur virus.

ToLCNDV-Tomato leaf curl New Delhi virus.

Having demonstrated the efficacy of RNA*later*^®^ as a robust tool for sampling and follow-up partial and full-length genome amplifications of begomoviruses, we performed specific PCR assays of 51 papaya leaf samples stored in RNA*later*^®^ for the begomoviruses ToLCBV, ToLCJoV, and ToLCNDV (Table 4). Three asymptomatic samples and three samples with PRSV-like symptoms tested negative for begomoviruses. PRSV-specific STNP and ELISA tests were also PRSV-negative in the asymptomatic samples, but positive for samples with PRSV-like symptoms. When the 45 samples with leaf curl symptoms typical of begomovirus infection were tested, however, ToLCJoV, ToLCBV, and ToLCNDV were detected, respectively, in 10, 29, and 4 of the samples (Table 4).

**Figure 6:**
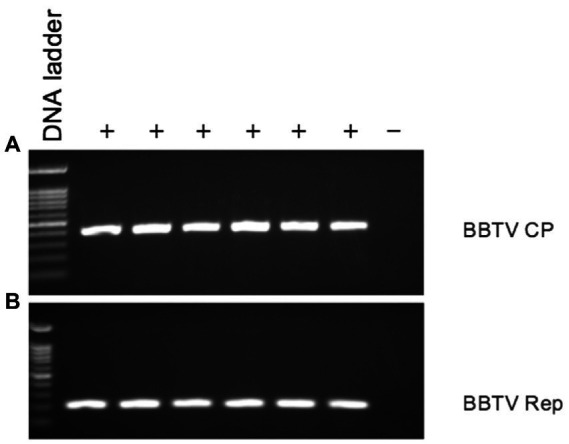
PCR analysis of symptomatic (+) and asymptomatic (−) banana leaf samples stored in RNA*later*^®^ for presence of BBTV using BBTV **(A)** CP gene-specific, and **(B)** Rep gene-specific primers.

DNA from six banana samples with symptoms of BBTV tested positive by PCR using BBTV-Rep-gene-specific primers, and CP-specific primers BBTV-HAF1 and BBTV-HAR1 (Hamim et al., 2017; Figure 6). Six PCR-positive samples also tested positive for BBTV by triple-antibody sandwich-ELISA using BBTV-specific antibodies.

## Discussion

Our research showed that RNA*later*^®^ technology can be used to transport and store leaf samples with virus-like symptoms from remote locations for extended periods before analysis for viral pathogens. Virus-infected papaya leaf tissue from the island of Oahu, Hawaii was preserved in RNA*late*r^®^ solutions for 7 to 30 days at room temperature until RNA extraction, followed by a three-day storage at 4°C, one-week storage at-20°C, and one-month storage at-80°C. Kamitani et al. (2016) used RNA*later*^®^ to prevent RNA degradation of *Arabidopsis halleri* leaf samples. Their samples were maintained in RNA*later* at 4°C for 1 day and then moved to-20°C for 1.5 months before RNA extraction (Kamitani et al., 2016; Honjo et al., 2020).

In our study, RNA yields obtained from RNA*later*^®^-stored virus-infected papaya leaves were reduced in quantity and quality after different storage times compared to fresh samples. From zero to 30 days, total RNA recovered from samples preserved in RNA*later*^®^ decreased gradually. Although the quantity of total RNA from RNAlater^®^-stored samples was reduced compared to fresh samples, the quality was sufficient for detection of plant viruses and amplification of their genomic sequences. Storage in RNA*later*^®^ is considered one of the best methods for preserving RNA for subsequent virus diagnosis (Rawiwan et al., 2021). According to Kohl et al. (2017), PCR and sequencing results from RNA*later*^®^-stored samples were equivalent to those from snap-frozen samples. Because of the additional equipment and costly transportation required, snap-freezing is often challenging in field situations. We have demonstrated that storage in RNA*later*^®^ solutions can overcome some of these limitations.

The full-length genomes of two PRSV isolates, BD-1 and BD-2, were identified by HTS in our study from composite RNAs pap-1 and pap-2, respectively. This indicates the effectiveness of storage in RNA*later*^®^ prior to whole-genome characterizations of plant RNA viruses. In Kenya, the presence of Moroccan watermelon mosaic virus (MWMV) and three different species of Carlaviruses in papaya tissues stored in RNA*later*^®^ was also determined using Illumina MiSeq^®^ next-generation sequencing (NGS) and validated using RT-PCR and Sanger sequencing (Mumo et al., 2020). Kamitani et al. (2016) used RNA-Seq to detect known viruses and identify novel viruses in RNA*later*^®^-preserved leaf samples of *Arabidopsis halleri* subsp. *gemmifera*. They identified infections by turnip mosaic virus, cucumber mosaic virus, and Brassica yellows virus. We demonstrated the use of RNA*later*^®^ technology for the routine diagnosis of plant RNA viruses and effectively detected distinct PRSV isolates BD-1 and BD-2 from papaya and DsMV from taro.

RNA*later*^®^-preserved plant material is suitable for the extraction of high-quality DNA and subsequent downstream analyses (LeFrois et al., 2016). In our studies, DNA extracted from papaya leaf samples stored in RNA*later*^®^ was used to detect and obtain complete or partial genomes of specific begomovirus species and their associated components by PCR and Sanger sequencing. In addition, we successfully detected BBTV from banana samples collected in Bangladesh and stored in RNA*later*^®^, confirming the results of Steward and Culley (2010) that showed RNA*later*^®^ could preserve the genomes of DNA viruses.

The successful immunodetection of plant viruses from material stored in RNA*later*^®^ supports the results of Reiser et al. (2011), who demonstrated that preserving samples in this reagent did not interfere with ELISA protein analysis of tissue extracts. In some cases, RNA*later*^®^ maintained the infectivity of enveloped and nonenveloped viruses for an extended time (Uhlenhaut and Kracht, 2005).

We demonstrated that plant viruses from leaf samples collected from a distant location and stored in RNA*later*^®^ for various lengths of time could still be detected by PCR, RT-PCR, STNP, Sanger sequencing, HTS and ELISA. The product allows fast and easy sampling of plant material in the field. The effective retrieval of RNA and DNA viral sequences from RNA*later*^®^-stored samples was demonstrated by different molecular techniques in this manuscript. The preservation of plant tissues at room temperature alleviates concerns about nucleic acid damage during storage. Importantly, all downstream analytical procedures were unaffected; no new protocol development was needed with RNA*late*r^®^.

Results obtained from studies of leaf samples from three different plant species stabilized in RNA*later*^®^, whether collected in Oahu, Hawaii, or brought to Hawaii from farmer’s fields in Bangladesh, were acceptable and reproducible among plants. In all cases, these samples yielded total RNA or DNA of a quality and quantity sufficient for plant virus detection using several methods. RNA*later*^®^ was used to preserve the papaya leaf tissues collected for this investigation from several districts of Bangladesh. Then, using specific RT-PCR or PCR tests, we were able to effectively identify, examine the distribution and variability of certain PRSV isolates and begomoviruses in the Bangladeshi papaya samples. Therefore, managing viral diseases in papaya orchards in Bangladesh can benefit from the identification and study of the distribution of viruses and specific virus isolates in RNA*later*^®^-preserved samples collected from Bangladesh. This technology provides a simple, sensitive, and specific tool for the diagnosis and molecular characterization of plant viral pathogens isolated from plant tissues. We conclude that this technology supports the advanced diagnostic methods used to identify viral pathogens infecting crop plants by allowing samples to be collected from remote areas and stored for extended periods.

## Data availability statement

The datasets presented in this study can be found in online repositories. The names of the repository/repositories and accession number(s) can be found in the article/supplementary material.

## Author contributions

IH and JH: conceptualization. IH, JH, and WB: methodology. IH: formal analysis, writing—original draft preparation, visualization, and investigation. IH, JH, WB, and MM: data curation. IH, JS, MM, MQ, and JH: writing—review and editing. JH and WB: supervision. All authors contributed to the article and approved the submitted version.

## Funding

This research was funded by the United States Agency for International Development as part of the Feed the Future initiative under the CGIAR Fund (award number BFS-G-1100002) and the predecessor fund of the Food Security and Crisis Mitigation II grant (award number EEM-G-0004-00013). The research was also funded by the USDA National Institute of Food and Agriculture, Hatch HAW09025-H (1001478), and the USDA-Agricultural Research Service (58–5320–4-012).

## Conflict of interest

The authors declare this research was conducted without any commercial or financial relationships that could be construed as a potential conflict of interest.

## Publisher’s note

All claims expressed in this article are solely those of the authors and do not necessarily represent those of their affiliated organizations, or those of the publisher, the editors and the reviewers. Any product that may be evaluated in this article, or claim that may be made by its manufacturer, is not guaranteed or endorsed by the publisher.

## Footnotes

^1^ https://www.ncbi.nlm.nih.gov/genome/viruses/

## References

[ref1] Al Rwahnih M. Rowhani A. Westrick N. Stevens K. Diaz-Lara A. Trouillas F.. (2018). Discovery of viruses and virus-like pathogens in pistachio using high-throughput sequencing. Plant Dis. 102, 1419–1425. doi: 10.1094/PDIS-12-17-1988-RE, PMID: 30673557

[ref800] Bateson M. F. Henderson J. Chaleeprom W. Gibbs A. J. Dale J. L. (1994). Papaya ringspot potyvirus: isolate variability and the origin of PRSV type P (Australia). J. General Virol. 75, 3547–3553., PMID: 7996146 10.1099/0022-1317-75-12-3547

[ref2] Bennike T. B. Kastaniegaard K. Padurariu S. Gaihede M. Birkelund S. Andersen V.. (2016). Comparing the proteome of snap frozen, RNA*later*® preserved, and formalin-fixed paraffin-embedded human tissue samples. EuPA Open Proteom. 10, 9–18. doi: 10.1016/j.euprot.2015.10.001, PMID: 29900094 PMC5988570

[ref4] Fleige S. Pfaffl M. W. (2006). RNA integrity and the effect on the real-time qRT-PCR performance. Mol. Asp. Med. 27, 126–139. doi: 10.1016/j.mam.2005.12.003, PMID: 16469371

[ref5] Hamim I. (2019). Molecular Analyses of Papaya Viruses in Bangladesh: Detection, Characterization, and Distribution (Doctoral dissertation, University of Hawai'i at Manoa), 103, 2920, 2924. doi: 10.1094/PDIS-12-18-2186-RE 31567059

[ref6] Hamim I. Al Rwahnih M. Borth W. B. Suzuki J. Y. Melzer M. J. Wall M. M.. (2019a). Papaya ringspot virus isolates from papaya in Bangladesh: detection, characterization, and distribution. Plant Dis. 103, 2920–2924. doi: 10.1094/PDIS-12-18-2186-RE, PMID: 31567059

[ref7] Hamim I. Borth W. Melzer M. J. Hu J. (2018). Ultra-sensitive detection of papaya ringspot virus using single-tube nested PCR. Acta Virol. 62, 379–385. doi: 10.4149/av_2018_405, PMID: 30472867

[ref8] Hamim I. Borth W. B. Melzer M. J. Suzuki J. Y. Wall M. M. Hu J. S. (2019b). Occurrence of tomato leaf curl Bangladesh virus and associated subviral DNA molecules in papaya in Bangladesh: molecular detection and characterization. Arch. Virol. 164, 1661–1665. doi: 10.1007/s00705-019-04235-8, PMID: 30949815

[ref9] Hamim I. Borth W. B. Suzuki J. Y. Melzer M. J. Wall M. M. Hu J. S. (2020). Molecular characterization of tomato leaf curl Joydebpur virus and tomato leaf curl New Delhi virus associated with severe leaf curl symptoms of papaya in Bangladesh. Eur. J. Plant Pathol. 158, 457–472. doi: 10.1007/s10658-020-02086-7

[ref10] Hamim I. Green J. C. Borth W. B. Melzer M. J. W. Hu J. (2017). First report of Banana bunchy top virus in Heliconia spp. on Hawaii. Plant Dis. 101:2153. doi: 10.1094/PDIS-02-17-0205-PDN

[ref11] Honjo M. N. Emura N. Kawagoe T. Sugisaka J. Kamitani M. Nagano A. J.. (2020). Seasonality of interactions between a plant virus and its host during persistent infection in a natural environment. ISME J. 14, 506–518. doi: 10.1038/s41396-019-0519-4, PMID: 31664159 PMC6976672

[ref12] Huang L. H. Lin P. H. Tsai K. W. Wang L. J. Huang Y. H. Kuo H. C.. (2017). The effects of storage temperature and duration of blood samples on DNA and RNA qualities. PLoS One 12:e0184692. doi: 10.1371/journal.pone.0184692, PMID: 28926588 PMC5604973

[ref13] Kamitani M. Nagano A. J. Honjo M. N. Kudoh H. (2016). RNA-Seq reveals virus–virus and virus–plant interactions in nature. FEMS Microbiol. Ecol. 92:176. doi: 10.1093/femsec/fiw176, PMID: 27549115 PMC5854034

[ref14] Kohl C. Wegener M. Nitsche A. Kurth A. (2017). Use of RNAlater® preservation for virome sequencing in outbreak settings. Front. Microbiol. 8, 1888. doi: 10.3389/fmicb.2017.01888, PMID: 29018436 PMC5623189

[ref15] Kruse C. P. Basu P. Luesse D. R. Wyatt S. E. (2017). Transcriptome and proteome responses in RNAlater® preserved tissue of Arabidopsis thaliana. PLoS One 12:e0175943. doi: 10.1371/journal.pone.0175943, PMID: 28423006 PMC5397022

[ref16] LeFrois C. E. Zhou M. Amador D. M. Sng N. Paul A. L. Ferl R. J. (2016). Enabling the spaceflight methylome: DNA isolated from plant tssues preserved in RNAlater® is suitable for bisulfite PCR assay of genome methylation. Grav. Space Res. 4, 28–37. doi: 10.2478/gsr-2016-0010

[ref17] Mäki A. Salmi P. Mikkonen A. Kremp A. Tiirola M. (2017). Sample preservation, DNA or RNA extraction and data analysis for high-throughput phytoplankton community sequencing. Front. Microbiol. 8, 1848. doi: 10.3389/fmicb.2017.01848, PMID: 29018424 PMC5622927

[ref18] Mumo N. N. Mamati G. E. Ateka E. M. Rimberia F. K. Asudi G. O. Boykin L. M.. (2020). Metagenomic analysis of plant viruses associated with papaya ringspot disease in *Carica papaya* L. in Kenya. Front. Microbiol. 11, 205. doi: 10.3389/fmicb.2020.00205, PMID: 32194518 PMC7064807

[ref20] Noa-Carrazana J. C. González-de-León D. Silva-Rosales L. (2007). Molecular characterization of a severe isolate of papaya ringspot virus in Mexico and its relationship with other isolates. Virus Genes 35, 109–117. doi: 10.1007/s11262-006-0039-y, PMID: 17082995

[ref21] Patel P. G. Selvarajah S. Guérard K. P. Bartlett J. M. Lapointe J. Berman D. M.. (2017). Reliability and performance of commercial RNA and DNA extraction kits for FFPE tissue cores. PLoS One 12:e0179732. doi: 10.1371/journal.pone.0179732, PMID: 28640876 PMC5480995

[ref22] Prendini L. Hanner R. DeSalle R. (2002). 11 obtaining, storing and archiving. Tech. Molecular System. Evol. 200:176. doi: 10.1007/978-3-0348-8125-8_11

[ref23] Rawiwan P. Khemthong M. Tattiyapong P. Huchzermeyer D. Surachetpong W. (2021). Effects of sample preservation and storage times on the detection of tilapia lake virus (TiLV) RNA in tilapia tissues. Aquaculture 533:736240. doi: 10.1016/j.aquaculture.2020.736240

[ref24] Reiser V. Smith R. C. Xue J. Kurtz M. M. Liu R. LeGrand C.. (2011). High-throughput simultaneous analysis of RNA, protein, and lipid biomarkers in heterogeneous tissue samples. Clin. Chem. 57, 1545–1555. doi: 10.1373/clinchem.2010.157743, PMID: 21914789

[ref900] Relova D. Rios L. Acevedo A. M. Coronado L. Perera C. L. Pérez L. J. (2018). Impact of RNA degradation on viral diagnosis: an understated but essential step for the successful establishment of a diagnosis network. Veterinary Sci. 5:19., PMID: 29415432 10.3390/vetsci5010019 PMC5876574

[ref25] Sreenivasulu M. Gopal D. V. R. (2010). Development of recombinant coat protein antibody based IC-RT-PCR and comparison of its sensitivity with other immunoassays for the detection of papaya ringspot virus isolates from India. Plant Pathol. J. 26, 25–31. doi: 10.5423/PPJ.2010.26.1.025

[ref26] Sirakov I. N. (2016). Nucleic acid isolation and downstream applications. Nucleic Acids-From Basic Aspects to Laboratory Tools, 1–26. doi: 10.5772/61833

[ref27] Steward G. F. Culley A. I. (2010). Extraction and purification of nucleic acids from viruses. Manual. Aqua. Viral Ecol. 16, 154–165. doi: 10.4319/mave.2010.978-0-9845591-0-7.154

[ref28] Uhlenhaut C. Kracht M. (2005). Viral infectivity is maintained by an RNA protection buffer. J. Virol. Methods 128, 189–191. doi: 10.1016/j.jviromet.2005.05.002, PMID: 15936833

[ref30] Wang D. Hamim I. Borth W. B. Melzer M. J. Sun G. F. Hu J. S. (2018b). First report of dasheen mosaic virus infecting taro (*Colocasia esculenta*) in Bangladesh. Plant Dis. 102:2668. doi: 10.1094/PDIS-03-18-0442-PDN

[ref31] Wang Y. Wu B. Borth W. B. Hamim I. Green J. C. Melzer M. J.. (2017). Molecular characterization and distribution of two strains of dasheen mosaic virus on Taro in Hawaii. Plant Dis. 101, 1980–1989. doi: 10.1094/PDIS-04-17-0516-RE, PMID: 30677375

[ref32] Wang M. R. Yang W. Zhao L. Li J. W. Liu K. Yu J. W.. (2018a). Cryopreservation of virus: a novel biotechnology for long-term preservation of virus in shoot tips. Plant Methods 14, 1–10. doi: 10.1186/s13007-018-0312-9 29942344 PMC5996562

[ref33] Yockteng R. Almeida A. M. Yee S. Andre T. Hill C. Specht C. D. (2013). A method for extracting high-quality RNA from diverse plants for next-generation sequencing and gene expression analyses. Appl. Plant Sci. 1:1300070. doi: 10.3732/apps.1300070, PMID: 25202509 PMC4103122

[ref34] Zheng L. Rodoni B. C. Gibbs M. J. Gibbs A. J. (2010). A novel pair of universal primers for the detection of potyviruses. Plant Pathol. 59, 211–220. doi: 10.1111/j.1365-3059.2009.02201.x

